# Primary Mediastinal Large B-cell Lymphoma

**DOI:** 10.1007/s11899-014-0219-0

**Published:** 2014-06-22

**Authors:** Anna Dabrowska-Iwanicka, Jan A. Walewski

**Affiliations:** Department of Lymphoid Malignancies, Maria Sklodowska-Curie Institute and Oncology Centre, 5 WK Roentgen Str, 02-781 Warszawa, Poland

**Keywords:** Primary mediastinal B-cell lymphoma, JAK-STAT pathway, Programmed death ligand (PD-L), Chemotherapy, Immunochemotherapy, Rituximab, Third-generation regimen, Radiotherapy, PET/CT, International prognostic index

## Abstract

Primary mediastinal B-cell lymphoma (PMBCL) is a relatively rare lymphoma subtype affecting mainly young adults. Its molecular signature and clinical features resemble classical Hodgkin lymphoma. The optimal chemotherapy for this lymphoma subtype has not been established. The addition of rituximab to anthracycline based chemotherapy improved response rates and survival. Many centers use R-CHOP as standard treatment, but the role of the intensified regimens and consolidation radiotherapy has to be clarified. Recent data coming from retrospective analyses and an ongoing prospective study addressing the problem of consolidation radiotherapy will help to better identify risk groups and apply risk-adapted and effective treatment strategies. The latest research has helped to understand molecular mechanisms of PMBCL pathogenesis and indicated targets of directed therapy for the future.

## Introduction

Primary mediastinal B-cell lymphoma (PMBCL) belongs to the group of aggressive diffuse large B-cell lymphomas. The current WHO 2008 classification distinguished this lymphoma as a separate entity due to its specific clinical and pathological features [1]. Gene expression profile studies showed that it shares common features with classical Hodgkin lymphoma (cHL) [20, 21]. Treatment outcomes in the pre-rituximab era were not satisfactory with high relapse rates. Adding rituximab to anthracycline based regimens improved patient prognosis, and R-CHOP has been widely adopted as the standard treatment. However, there are still unresolved questions in the therapy of PMBCL. There are questions such as, is R-CHOP an optimal regimen for all patients? Historical data indicated superiority of more intense chemotherapy regimens, but they have not been compared to R-CHOP directly, and there is no consensus which group of patients would benefit from intensified regimens. Also, there is no convincing data supporting the use of radiotherapy. Retrospective studies generally did not show survival benefit, and late toxicities like cardiotoxicity and secondary cancers cannot be neglected. However, the questions if radiotherapy can be safely omitted and in which patient group it can be omitted, so far have not been answered. Relatively low patient numbers are the main obstacle in conducting randomized prospective trials, so therapeutic decisions have been based mainly on retrospective studies.

## Epidemiology

PMBCL constitutes approximately 2 % to 4 % of all non-Hodgkin lymphomas (around 6 % of diffuse large B-cell lymphomas (DLBCL)). This disease affects mainly young adults (median age of 35), predominantly women (female/male ratio 1.7-2/1) [2]. There are also cases of PMBCL among children and adolescents [3]. No risk factors for this type of lymphoma have been identified; however, a familial case of PMBCL has been described in Finland, probably related to the 5533C > A mutation in the MLL gene. [4].

## Clinical Presentation

PMBCL typically presents as a large, fast-growing tumor with invasion usually limited to the anterior-upper mediastinum although it tends to infiltrate adjacent thoracic structures like the chest wall, pleura, lungs, pericardium, and heart causing pleural/pericardial effusion in approximately 30–50 % of cases. The disease is mainly locally advanced. Eighty percent of patients have clinical stage I and II and 75 % of them have bulky disease with a tumor mass exceeding 10 cm. Enlarged lymph nodes localized outside the mediastinum are rarely found. Bone marrow infiltration is seen in few cases [5, 6, 7••, 8]. Recurrent disease tends to spread to distant extranodal organs like the kidneys, adrenal glands, liver, central nervous system, and less frequently to the lymph nodes. Typical symptoms such as cough, tachypnoe, vein thrombosis, chest pain, or dysphagia are related to the tumor mass infiltration or compression, with a history of complaints for usually less than three months. Approximately half of the patients present with upper vena cava syndrome. Systemic symptoms, mainly weight loss and fever, are relatively rare and they affect less than 20 % of patients [2].

## Pathology

PMBCL arises in the thymus from a so-called thymic B-cell originating either from a germinal center or a nongerminal center but with an expression of an activation induced cytidine deaminase (AID) gene. Cells are heterogenous, medium-sized to large-sized, with a pale, abundant cytoplasm. Their nuclei also show a degree of heterogeneity. They could be oval, irregular, pleomorphic like Reed-Sternberg cells or multilobated like in DLBCL. Characteristic feature of PMBCL is sclerosis dividing tumor tissue into compartments. Collagen bands are fine and not as broad as in cHL nodular sclerosis (NS) types. Immunophenotyping of tumor cells shows positive surface staining for pan-B cell antigens: CD19, CD20, CD22, also in a majority of cases for CD23, CD45 and MAL [1, 9, 10]. The last, associated with the specific population of B lymphocytes, thymic medullary B-cells, is expressed in 70 % of PMBCL, and it might be useful in differentiating PMBCL from DLBCL (expression only in 3 %) [11, 12]. CD30, being an attractive therapy target in Hodgkin lymphoma or anaplastic lymphoma, is also positive in most of the PMBCL cases but its expression is usually weaker than in the mentioned lymphoma types. PMBCL cells are usually CD10(-) and CD15(-). They also typically lack surface immunoglobulin (sIg) and, what is characteristic for this lymphoma type, only one component of the B-cell receptor is present, namely CD79a [10]. Another significant feature of PMBCL cells is decreased or, in some cases, absent expression of human leukocyte antigen class I and II (HLA) correlated to worse outcome [13]. MUM1/IRF4 expression has been found to be present in 45 % of the cells and is also associated with inferior survival [14]. Staining for transcriptional factors PU.1, OCT2, PAX5, and BOB1 is often positive [10]. BCL2 and BCL6 are positive by immunohistochemistry in a majority of cases [10, 15, 16]. Knowledge of the characteristic immunophenotype of PMBCL cells is necessary in differential diagnosis. A decision tree for discriminating between PMBCL and cHL has been proposed by Hoeller S. et al., using mainly three markers: CD79a or alternatively BOB1 with combination with cyclin E. Applying this diagram enables classification correctly for 97-98 % of the patients to either PMBCL or cHL groups [17].

## Molecular Pathology

Somatic hypermutations of immunoglobulin genes and BCL6 in PMBCL were found in some studies and they confirmed germinal (post-germinal) center origin of the malignant cells [18]. Notably, the mutated region in BCL6 gene differs from DLBCL and follicular lymphoma [19]. In 2003 two independent studies were published, indicating that PMBCL has a unique molecular transcriptional pattern, distinct from DLBCL but having common features with cHL [20, 21]. Gene expression profiling (GEP) showed that over one-third of the genes overexpressed in PMBCL are also strongly expressed in HL cells. These genes are mostly related to the important signaling pathways of NFκB and JAK-STAT [21]. Dysregulation of these pathways contributes to the malignant phenotype of PMBCL leading to the inhibition of apoptosis and increased proliferation [22••]. NFκB is one of the most important transcription factors in the cell, responsible for proliferation and survival and constitutive activation of NFκB is typical for PMBCL [23]. This is achieved via different mechanisms such as overexpression of the tumor necrosis factor (TNF) receptor superfamily activating this pathway and overexpression of NFκB complex members [22••]. A20 protein is a negative regulator of IκB and NFκB, and a gene encoding this protein, TNFAIP3, is mutated in 36 % of cases resulting in constitutive activation of NFκB [24]. Nuclear localisation of REL, one of the NFκB complex proteins, is related to the activation of the NFκB pathway. Genomic gains and amplifications of the REL protooncogene locus on the short arm of chromosome 2p are present in over half of the PMBCL cases and associated with the nuclear position of REL [25, 26]. JAK-STAT is another major pathway responsible for the regulation of cell proliferation. The JAK-STAT signaling cascade is activated by interleukin (IL) receptors, mainly IL-4 and IL-13, and aberrations have been found on different levels of this pathway, starting from an enhanced IL-13 receptor expression [20, 21]. Genomic gains of distant regions of chromosome 9 - 9p24.1 containing a locus for JAK2 have been found in approximately 63 % of PMBCL cases, and they correlate with an increased JAK2 mRNA and protein levels [27•], leading to JAK-STAT cascade activation and cell proliferation. Amplification of 9p24.1 and overexpression of JAK2 is also associated with an upregulation of an immunoregulatory programmed death (PD)-1 ligand resulting in the exhaustion of T lymphocytes [27•]. One of the key proteins in the JAK-STAT pathway is STAT6 protein and constitutive activation of STAT6 is typical for PMBCL [28]. Moreover, in 36 % of PMBCL somatic mutations in STAT6 DNA-binding domain cases have been identified, confirming the role of dysregulations of JAK-STAT in PMBCL pathogenesis [29]. Genes coding for a suppressor of cytokine signaling 1 (SOCS-1), a negative regulator of this pathway, have been found to be mutated in some PMBCL patient cases and in mediastinal B-cell lines leading to delayed degradation and prolonged phosphorylation of JAK2 [30]. PTNP1 being another negative regulator of JAK-STAT has been recently described to be mutated in 22 % of PMBCL cases, also resulting in increased phosphorylation of JAK2, although only a trend towards inferior progression-free survival in mutated cases was noted [31].

Another pathological mechanism giving survival advantage is correlated to tumor microenvironment reactions enabling the tumor to escape from immunosurveillance. One of them is a decreased expression of major histocompatibility complex (MHC) class II genes and proteins in PMBCL as shown by gene expression profiling and immunohistochemistry studies, correlating with reduced cytotoxic CD8 T cell numbers and inferior survival [13, 32]. MHC expression is regulated by the MHC class II transactivator *CIITA.* Genomic breaks in *CIITA* were found to be present in 38 % of PMBCL, and they were associated with lower levels of MHC class II expression, and a significantly lower patient survival rate [33]. Genomic rearrangements in *CIITA* also have an impact on the expression of programmed cell death ligands-1 – PD-1 ligands on the surface of PMBCL cells (PD-L2 - CD273 and PD-L1- CD274). These molecules are involved in costimulatory signal transduction between malignant cells and PD-1 receptor on T cells, in addition to T cell receptor (TCR) signaling, modulating T cell activity. Overexpression of PD-L2 and PD-L1 leads to an exhaustion of infiltrating T cells and the tumor’s escape from immunosurveillance [33]. Genes encoding PD-L1 and PD-L2 are located in the 9p24.1 region, similarly to JAK2 [27•]. Recently, genomic rearrangements involving PD-L locus have been described in 20 % PMBCL, including break-apart, amplifications, and gains, and they were associated with PD-L protein overexpression. It was noteworthy that, although no survival correlation was established between rearranged and nonrearranged cases, the PD-L levels in PMBCL cells were higher than in the normal control, indicating another regulation pathway of PD-L expression [34]. Enhanced expression of PD-L1 on PMBCL and on tumor associated macrophages was also confirmed by the immunohistochemistry method in 71 % malignant cells [35•].

The results of cited studies shed light on the biology of PMBCL and some of the dysregulated molecular mechanisms described above will become an attractive therapy target in the future. Preclinical studies show that selective inhibition of JAK2 with fedratinib reduced phosphorylation of JAK2 and other proteins from the STAT family in cHL and PMBCL cell lines. It also inhibited the expression of PD-L1. In murine models inhibition of JAK2 significantly decreased tumor growth and prolonged survival, which was correlated with reduced STAT3 expression [36••]. PD-L1 has already become the subject of clinical trials in many cancers giving response rates in 20-25 % of patients [37] and seems to be also a natural therapy target in lymphomas overexpressing PD-L.

## Diagnosis and Differential Diagnosis

Typical localization of PMBCL confined to mediastinum without the involvement of peripheral lymph nodes requires more invasive diagnostic procedures. Mediastinoscopy, anterior mediastinotomy, or percutaneous CT-guided core needle biopsies, are usually performed [2, 15]. Representative and relatively extensive tissue samples should be taken, as the cells can be damaged during the biopsy, which can make diagnosis more difficult to establish.

Differential diagnosis of PMBCL includes other types of lymphomas with mediastinal localization [9]:

- “gray zone” lymphoma - B-cell lymphoma, unclassifiable, with features intermediate between diffuse large B-cell lymphoma and classical Hodgkin lymphoma [38],
- composite lymphoma consisting of two types of lymphoma: PMBCL and a cHL
- mediastinal sequential lymphomas (i.e., PMBCL relapsing as HL)
- diffuse large B-cell lymphoma with anterior mediastinum involvement
- cHL NS type [38]
- T-cell lymphoblastic lymphoma
- Thymoma
- Germ cell tumors
- Metastatic carcinomas

Diagnosis is made on the basis of histopathological examination with mandatory immunohistochemical staining and typical clinical presentation.

Diagnostic procedures performed to assess clinical stage are typical and include physical examination, whole-body computer tomography, bone marrow biopsy, whole blood count, and blood biochemistry. Elevated LDH is found in 70-80 % of cases, and it can often be the only laboratory abnormality [6, 8]. β2-microglobulin is usually within the normal range [39]. PMBCL belongs to FDG-avid lymphomas, so PET/CT has been incorporated into the diagnostic procedures carried out before and at the end of therapy (EOT) [40]. Clinical stage is assessed using the Ann Arbor staging system, and, as mentioned above, about 75 % of patients have clinical stage (CS) I or II [5, 6, 7••, 8].

## Prognostic Factors

For the assessment of risk category, a standard international prognostic index (IPI) is used. However, its role in PMBCL is limited due to the fact that two out of five risk factors determining patients’ survival are generally not present: age above 60 and CS III or IV [5, 6, 7••, 8]. Another reason for conflicting results of using IPI for assessing patient risk is the lack of consistency in evaluating the stage of the disease. Mediastinal tumor infiltrating per continuum/extension other thoracic structures can be described by a treating physician as stage IIE or stage IV. This may lead to discrepancies in evaluating the role of IPI in PMBCL in retrospective analyses published by different centers. In the authors’ opinion as described by Vassilakopoulos et al. [41•], CS IV should be confined to lymphoma with extensive dissemination to extranodal organs, whereas a tumor infiltrating to a limited degree adjacent thoracic structures such as pleura, pericardium, thoracic wall or lungs should be assessed as CS IIE. In the retrospective analysis from 2012, age-adjusted IPI (aaIPI) in a multivariate analysis failed to be of prognostic significance [41•]. This was similar to an earlier series of 141 patients from Memorial Sloan-Kettering Cancer Center (MSKCC) [8]. In various published series of PMBCL patients, other negative prognostic factors for survival were described: age over 40 years of age, CS III and IV, bulky disease, male sex, poor performance status (PS), and LDH > 2 x UNL [5, 6], but their prognostic power has not been validated in large, prospective studies in the rituximab era. However, IPI proved to be a significant factor for survival in R-CHOP-treated patients in a recent retrospective study by a Japanese multicenter study group including 345 patients (187 of them treated with the R-CHOP regimen) [7••]. Multivariate analysis showed a pleural/pericardial effusion to be another adverse factor for PFS and a novel prognostic model for PMBCL (PMBL prognostic index – PMBIPI) was designed, including two factors: high/intermediate-risk and high-risk IPI and the presence of a pleural/pericardial effusion. Patients in a low-risk group (0 factors) had a favorable overall survival (OS) of 97 % and progression-free survival (PFS) 89 % at four years, whereas patients with two adverse risk factors had a statistically inferior 4-year OS and PFS of 72 % (*P* = 0.001) and 44 % (*P* < 0.001). As the authors stated, this novel prognostic index needs to be evaluated in prospective studies [7••].

## First-Line Therapy

Initial treatment is critical in the management of PMBCL since outcomes of second and further therapy lines in relapsed/progressive disease are unsatisfactory [6, 42, 43]. R-CHOP is used in many centers world-wide but there are some reports on the better efficacy of more intense chemotherapy regimens (so-called third-generation) with a higher cytostatics dose/density. However, these analyses are mainly retrospective and based on a small number of patients. The role of consolidation radiotherapy in the rituximab era and also in conjunction with third-generation chemotherapy also needs to be determined.

Historically, before the rituximab era, PMBCL patients, like in DLBCL, were treated with CHOP, usually with consolidation radiotherapy, achieving a complete remission (CR) rate of 48-70 % and a long-term overall survival of 33-65 % [5, 8, 44] (Tables 1 and 2). A MInT study comparing CHOP to R-CHOP is one of few prospective trials recruiting PMBCL patients, who constituted 11 % (*n* = 87) of all 824 DLBCL patients [45••]. The study was limited to young (age < 60), low-risk patients, with CS II-IV or I bulky and with IPI ≤1. The analysis showed that in the PMBCL sub-group, the addition of rituximab to six cycles of CHOP-like therapy [mainly CHOP-21 (50 %) and CHOEP-21 (49.5 %)] significantly increases CR rates (84 % vs 50 %; *P* = 0.03), lowers progression rates (2.5 % *vs* 24 %; *P* = 0.006), prolongs three year event-free survival (3-year EFS) - 78 % *vs* 52 %; *P* = 0,012 and 3 year-OS (88.5 % vs 78.2 %; *P* = 0.158). The difference in OS was not statistically significant for the PMBCL group in contrast to DLBCL (probably due to the smaller number of patients). In a multivariate analysis therapy containing rituximab and the absence of bulky disease were significant positive prognostic factors for OS, EFS, and overall response rates (ORR). The results of the MInT study, clearly showed that, in low-risk PMBCL, adding rituximab to CHOP improves treatment outcome, mainly by decreasing the progression rate, which established R-CHOP regimen as a standard of care in many centers. Many retrospective reports comparing R-CHOP with CHOP confirmed good outcomes in R-CHOP treated patients yielding 5-year PFS 68 – 77 % and 5-year OS 79-90 %; some of them including a relatively high number of patients (Table 2) [7••, 41•].

**Table 1:** Selected studies in PMBCL applying intensified regimens published after year 2000. Numbers in brackets refer to patient numbers in different chemotherapy groups (column 4) and to their outcome (OS, PFS) – columns 8, 9. All studies are retrospective, if not otherwise specified

[Ref]	Year (data collection)	Setting	Number of patients	High IPI (%)	CHTH	RTH	OS	PFS
[5]	2002 (1981-1999)	Multicenter	426 (105/277/44)	21	CHOP(-like)//3rd-generation//HDT/SCT	Yes in 84 % pts	10-y 65 % (44/71/ 77 %)	10-y 62 % (35/67/78 %)
[42]	2004 (1982-1999)	Multicenter	138 (43/95)	22	CHOP//MACOP-B( VACOP-B)	Yes in 75.5 % pts in CR	N/D	5-y EFS 39.5 %/75.7 %
[8]	2005 (1980-1995)	Single center	141(56/68/17)	28	CHOP(-like)//NHL-15//HDT/SCT	Yes (only in 23 %)	10.9-y 66 % (51/84/78 %)	10.9-y 50 % (34/60/60 %)
[6]	2006 (1980-2003)	Population study	153 (63/47/18*)	N/D (only aaiPI)	CHOP(-like)//MACOP-B(VACOP-B)//R-CHOP	Depending on recommendations	5-y 75 % (71/87/81 %)	5-y 69 % (N/D)
[65]	2008 (1992-2006)	Single center	68(42/26)	26 %	CHOP/3rd generation	Yes in 87 %	5-y 61 % (50/91 %)	5-y 52 % (33/83 %)
[56]	2010 (N/D)	Single center	54	N/D	R-CHOP/ICE	No	3-y 88 %	3-y 78 %
[51•]	2011 (2002-2010)	Single center prospective	15	N/D	GMALL B-ALL/NHL	Yes in 67 %	5-y 100 %	5-y 93.3 %
[49••]	2013 (1999-2012)	Single prospective	51	N/D	R-DA-EPOCH	No per protocol	5-y 97 %	5-y EFS 93 %
[52]	2011 (2004-2009)	Single center**	42	45	GMALL B-ALL/NHL	Yes in 89 % ***	5-y 98 %	5-y 93 %

Chemotherapy regimens described in references

Abbreviations: HDT/SCT – high dose therapy and stem cell transplantation, CHTH – chemotherapy, N/D – no data

Other abbreviations as in the text

*Numbers do not sum up to 153 since some patients received elderly regimens and not all were included in survival analysis

**Data extrapolated from multicenter analysis comparing different therapy regimens. Patients treated with an intense-chemotherapy protocol were treated in a single center

***Data concerning RTH in therapy subgroups was not published in the abstract (personal communication)

**Table 2:** Selected studies in PMBCL comparing CHOP to R-CHOP published after year 2000. Numbers in brackets refer to patient numbers in different chemotherapy groups (column 4) and to their outcome (OS, PFS) – columns 8, 9. All studies are retrospective, if not otherwise specified

[Ref]	Year (data collection)	Type of study	Number of patients	High IPI (%)	Chemotherapy	RTH	OS	PFS (EFS, TTP)
[45••]	2011 (N/D)	Prospective randomized	87 (43/44)	0	CHOP/R-CHOP (-like)	Yes in 67/71 %	3-y 83 % (78/89 %)	3-y EFS 65 % (52/87 %)
[41•]	2012 (N/D)	Multicenter	111 (76/45)	22/29	CHOP/R-CHOP	Yes in 52/76 % CR pts	5-y (69/89 %)	5-y EFS (47/80 %)
[58•]	2012 (N/D)	Population study	176 (80/96)	N/D	CHOP/R-CHOP	Yes variable(PET-guided****)	5-y (70/88 %)	5-y TTP (65/78 %)
[7••]	2013 (1986-2012)	Multicenter	345(44/187/45/57)	48	CHOP//R-CHOP//R-DA-EPOCH//2/3rd generation regimens//HDT + SCT	Yes in 42 % pts	4-y 87 % (67/90/100/91/92 %)	4-y PFS 70 % (40/71/100/83/76 %)
[46•]	2013 (1996-2011)	Single center	63	33	R-CHOP	Yes in 77 % pts	5-y 79 %	5-y PFS 68 %
[64]	2013	Single center	79	52 (aaIPI > 1)	CHOP/R-CHOP	Yes in 76 % pts	5-y 62 % (48/84 %)	5-y PFS 59 % (44/77 %)

Abbreviations: HDT/SCT – high dose therapy and stem cell transplantation, CHTH – chemotherapy, N/D – no data

Other abbreviations as in the text

****Patients were treated with RTH depending on era – earlier cohort per protocol, later – RTH was PET-guided

Despite the improvement of treatment outcomes with the introduction of rituximab, there is a subgroup of patients relapsing on primary R-CHOP chemotherapy. In the cited paper by Vassilakopulous, patients with aaIPI ≥ 2 had a worse survival of 5-year FFP 63 % and 5-year OS 75 % [41•]. Similarly, in the above mentioned multicenter analysis from Japan, patients with two adverse risk factors had a statistically inferior 4-year PFS and OS of 44 % and 72 % [7••]. Another group reported on the high rate of treatment failure in 63 R-CHOP patients, including 33 % with high/intermediate- and high-risk IPI [46•]. 5-year PFS was 68 % and 5-year OS was 79 % for the whole population; however, 21 % of patients had primary refractory disease or relapsed early (8 %) and 63 % of them died of lymphoma progression. Adverse prognostic factors for treatment failure were: age > 60, numerous extranodal sites, CS > II in multivariate analyses, and also aaIPI in a univariate analysis.

**Fig. 1:**
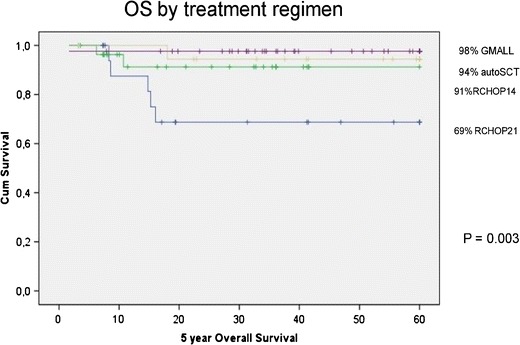
Survival of PMBCL patients treated with different chemotherapy regimens – retrospective analysis of the Polish Lymphoma Research Group. Treatment outcomes of patients treated with different chemotherapy regimens: standard R-CHOP-21, dose-dense (R-CHOP-14), and dose-intense (GMALL B-ALL/NHL 2002 protocol and HDT/autoSCT) regimens

Limited efficacy of R-CHOP in poor risk patients has prompted some groups to use more intense chemotherapy regimens. Such treatment approaches were applied in the pre-rituximab era, resulting in significantly better outcomes as compared to standard CHOP. One of the largest studies was a multinational retrospective analysis including 426 patients, which proved the superiority of the third-generation regimens (mainly MACOP-B and VACOP-B) over CHOP and CHOP-like [5]. Similar results were reported by other study groups [8, 42, 47], (Table 1). There is no certainty if adding rituximab to the third-generation chemotherapy brings any clinical benefit. Comparing the retrospective data of two groups of patients, that is, those treated with MACOP-B/VACOP-B and those with the same regimen but with the addition of rituximab, showed no statistical difference in relapse free survival and CR rates [48]. A more important problem that has not been addressed in randomized trials is the evaluation of third-generation regimens in the rituximab era by comparing them to standard R-CHOP chemotherapy. However, some papers on that topic have been published. They are mainly retrospective analyses or single-arm phase II studies assessing treatment outcomes with the use of more intense regimens. In 2013 the results of a prospective, phase II trial carried out in the National Cancer Institute (NCI) were published. Fifty-one patients with PMBCL were treated with 6-8 cycles of rituximab dose adjusted EPOCH (R-DA-EPOCH) chemotherapy without consolidation radiotherapy. It was given to only 4 % of patients who did not achieve CR [49••]. Adverse prognostic factors were frequently present in this population. Five-year EFS was 93 % and 5-year OS was 97 % with a median follow-up of 63 months. Toxicity was mainly hematological with neutropenia grade 4 occurring in 50 % of cycles, thrombocytopenia grade 4 in 6 %, and neutropenic fever in 13 % cycles. The results were compared to the historical cohort of DA-EPOCH-treated patients and, in contrast to Zinzani’s study, significant survival benefit for the rituximab-containing regimen was proved. Another intensified chemotherapy regimen is GMALL B-ALL/NHL 2002, based on pediatric ALL protocols, created by the German Multicenter Adult ALL Study Group (GMALL). It is an intense, multidrug regimen consisting of six courses, each containing rituximab and methotrexate at a dose of 1.5 g/m^2^, with intrathecal prophylaxis. At first, radiotherapy was obligatory but after protocol amendment it was left to the physician’s discretion, depending on the response assessment. The first results on 44 patients treated (without rituximab but with consolidation radiotherapy) in a few German centers were published in 2002 and they showed ORR exceeding 90 %, PFS – 85 % and OS – 82 % [50]. In 2011 the results of the next cohort of 15 patients treated with rituximab confirmed the high efficacy of this regimen yielding 5-year PFS 93.3 %, and 5-year OS 100 % [51•]. Our own experience in applying this intense chemotherapy regimen is also very encouraging. Since 2004 we have treated over 65 PMBCL patients including those with negative prognostic factors. With a median follow-up of 41 months 5-year PFS was 92 % and 5-year OS was 93 %. Most of the patients received consolidation radiotherapy. Toxicity was manageable and similar to previously published results: neutropenia 3 and 4 grade in 81 % of all cycles, trombocytopenia grade >2 in 58 % of the cycles, neutropenic fever in 28 % of the cycles, and also mucositis was noted in 27 % of the cycles. A retrospective survey of 109 patients by the Polish Lymphoma Research Group showed that patients treated with R-CHOP-21 had a significantly higher risk of treatment failure as compared to dose dense (R-CHOP-14) and dose intensified (GMALL B-ALL/NHL 2002 and high-dose therapy consolidation) groups [52] (Fig. 1). Although intensified regimens like R-DA-EPOCH and GMALL B-ALL/NHL 2002 seem to improve survival, their superiority over R-CHOP has not been proved in randomized trials and this potential benefit must be balanced against significant, albeit manageable, toxicity.

## Evaluation of Response to Therapy – The Role of PET/CT

The most useful, although not ideal tool, for assessing response to treatment is PET/CT [40]. Earlier CT was used but its value was sometimes limited as in many cases residual tumors were present after the treatment due to excessive fibrosis, and distinguishing between active and non-active mass was impossible. Published data show that PMBCL PET/CT has high sensitivity and high negative predictive value (NPV) but low specificity and positive predictive value (PPV) [54•, 55]. In the NCI series, 18 out of 36 patients with residual mass had positive PET but only three with SUV ≥5 had active disease [49••]. In an Italian cohort of 37 patients, 68 % had positive PET after R-chemotherapy but after radiotherapy 85 % converted to CR [53]. Only patients with the highest score of 5 progressed despite radiotherapy and their 2-year OS was significantly worse (33 % vs 100 %). Another important study recently published was IELSG-26 by the International Extranodal Lymphoma Study Group [54•]. There were 115 patients assessed with PET/CT after immunochemotherapy. Forty-seven percent achieved metabolic CR (metCR) (^18^FDG uptake below mediastinal blood pool, score 1 and 2) and had an excellent 5-year OS of 100 % and PFS 98 %. Among 53 % PET-positive patients those with score 4 and 5 (^18^FDG uptake above liver) had significantly worse 5-year PFS (68 % vs 99 %) and OS (83 % vs 100 %) than patients with score 1-3. None of the patients with score 3 (residual uptake) progressed/relapsed, but notably 89 % of them received radiotherapy, so the rate of real false positive results in this group is unknown. This study confirms good prognosis of EOT-PET-negative patients and a high proportion of positive EOT-PET in PMBCL, probably due to the inflammation or thymic rebound contributing to relatively low PPV of PET. Consistent with previous studies, patients with the highest scores/^18^FDG uptakes are at the highest risk of progression/relapse [49••, 53, 54•]. Data on the role of interim PET/CT is rather scarce. In a study by MSKCC, interim PET did not prove to be of prognostic value [56]. However, two recent reports confirmed high NPV (86-100 %) and lower PPV (30-75 %) of interim PET and in both studies patients with negative interim PET achieved long-term remission [55, 57].

## The Role of Consolidation Radiotherapy

In the past, radiotherapy was an important part of the treatment strategy, mainly due to the less effective chemotherapy in the pre-rituximab era, but no clear survival benefit was proven. Retrospective analysis of 426 patients showed that involved-field radiotherapy (IFRTH) increased CR rates in both standard and intense chemotherapy groups. Eighty-one percent of patients with PR converted to CR, however, no improvement in survival was observed [5]. In another study, patients who received radiotherapy had significantly longer EFS, regardless of their chemotherapy regimen, with no difference in OS [42]. In a population based analysis from 2006, no difference in PFS or OS between irradiated and nonirradiated patients treated with various chemotherapy regimens was observed [6]. Other studies with more intense treatment regimens showed good results without IFRTH [8, 47]. With the advent of more effective chemotherapy yielding high CR rates, the necessity of conducting IFRTH has become a matter of debate. In the cited NCI study, 9 6 % of patients treated with R-DA-EPOCH were not irradiated and achieved 5-year OS of 97 % [49••]. The MSKCC group showed 3-year OS 88 % and 3-year PFS 78 % for 54 patients treated with R-CHOP/ICE without radiotherapy [56]. A PET-guided strategy proposed by the British Columbia Cancer Agency indicated that the outcomes of patients with negative EOT-PET, who did not receive IFRTH, were similar to irradiated EOT-PET positive patients, suggesting that achieving metabolic CR may reduce the need for radiotherapy [58•]. Similar conclusions were drawn from the retrospective analysis from Italy. Radiotherapy was given only to PET-positive patients and there was no difference in disease free survival between this group and the PET-negative nonirradiated group [59]. In a Japanese study, the outcomes of PET-negative patients were similar, irrespective if they received IFRTH or not [7••]. A newly launched IELSG-37 study will probably give a definite answer to the question on the role of consolidation radiotherapy in PMBCL. The study is powered to determine noninferior outcome in patients not receiving radiotherapy. Patients who achieve metCR after R-CHOR/R-CHOP-like chemotherapy (R-CHOP-14/21, R-MACOP/B, R-DA-EPOCH, R-ACVBP) will be randomized to mediastinal radiotherapy of 30 Gy or to the observation arm. This important trial will help to determine whether omitting IFRTH, which allows sparing late toxicity in this young patient population, is a safe strategy [www.clinicaltrials.gov; NCT01599559].

## Treatment of Relapsed/Refractory Disease. The Role of Stem Cell Transplantation

In the pre-rituximab era, early progression was a relatively frequent event and occurred in approximately 20 % of cases. Immunochemotherapy significantly decreased this rate [45••] but primary refractory or relapsed disease still remains a problem, especially in the high-risk population. Relapses usually occur early, within 12-24 months, very rarely beyond two years, typically affecting extranodal sites, including the central nervous system (CNS). Earlier data on the risk of CNS relapses indicated quite a high risk of CNS involvement at relapse at up to 27 % [60], but recent publications do not confirm these numbers. Isolated CNS relapse was described in 2 % of 100 R-CHOP +/- RTH-treated patients and in 4.4 % of 45 CHOP-treated patients [61]. A British Columbia study also shows a low incidence of CNS relapse in similar patient subgroups (2.1 % for R-CHOP vs 3.2 % for CHOP) [58•]. Treatment strategies for relapsed disease do not differ from DLBCL salvage therapy including those not cross-resistant agents and consolidation with high-dose therapy and stem cell transplantation (SCT), mainly autologous, for fit patients; however, the results are rather disappointing. A retrospective survey from 2008, comparing 37 relapsed/refractory PMBCL patients with 143 DLBCL patients, showed that in PMBCL patients ORR to first-line salvage therapy was lower as compared to DLBCL (25 % vs 48 %, *P* = 0.01) and 2-year OS was also inferior (15 % vs 34 %, *P* = 0.018) [43]. More PMBCL patients progressed after salvage therapy (61 % vs 35 %) and fewer patients achieved remission and proceeded to autologous SCT. PMBCL patients with chemosensitive disease had similar outcomes to DLBCL patients after autoSCT. Primary refractory patients had the worst prognosis.

Autologous SCT is also used as a consolidation of the first line therapy, especially in high-risk patients and in patients who achieved only PR. In the GEL-TAMO experience, patient outcome depended mostly on the disease status before autoSCT. Patients in CR had 4-year PFS and OS of 81 % and 84 %, patients in PR 56 % and 64 %, respectively [62]. Primary refractory patients had significantly inferior survival with 4-year PFS of 16 % and OS of 23 %. Similar results were reported by other groups [63]. In a retrospective analysis of PLRG, no difference in survival was found between intensified GMALL B-ALL/NHL 2002 regimen and frontline treatment with autoSCT after R-CHOP chemotherapy [52], similarly to the IELSG cohort [5].

In summary, autoSCT remains a valid strategy in relapsed/refractory disease; however, the main factor determining long-term survival is achieving CR before the procedure. Since rituximab based chemotherapy and third-generation regimens improved survival in PMBCL, the role of frontline autoSCT is now debatable.

## Conclusions

PMBCL is a distinct clinicopathological entity and needs to be thoroughly differentiated with other lymphoma types, especially medDLBCL, cHL and gray zone lymphoma. Since the incidence of PMBCL is not high, the optimal treatment choice is based mainly on retrospective data or few prospective studies without control groups or including low patient numbers. There is a strong rationale supporting the use of rituximab based chemotherapy coming from a subgroup analysis of a prospective MInT trial and from numerous retrospective analyses with historical control groups. The results unequivocally show that the addition of rituximab to a standard anthracycline containing regimen CHOP/CHOP-like improves response rates, survival, reduces progression rates, and, therefore, R-CHOP has become a new standard of care in many centers. Despite this improvement, the risk of disease progression/relapse is still not negligible. It is up to 20 %, especially in high-risk groups, and intensified regimens are being developed and used. These regimens seem to bring clinical benefits in survival, allowing in some cases to omit IFRTH, but at the cost of significantly increased toxicity. More importantly, they have not been compared to R-CHOP in a randomized study. As the substantial part of patients achieve long-term survival with R-CHOP (+/- RTH), the main problem is to identify a group of patients who would really benefit from the third-generation regimes and for whom this excessive, though short-term toxicity would be clinically justifiable. The role of radiotherapy in the era of more effective immunochemotherapy remains a matter of debate. It undoubtedly improves the quality of response, but there is no consensus in which clinical settings radiotherapy can be safely omitted. A PET-guided approach based on data from retrospective surveys seems to be the most rational one, and the ongoing prospective randomized study will hopefully answer this important clinical question. Response to standard salvage therapy in refractory/relapsed disease is lower than in DLBCL and new treatment strategies are needed. Recent research has brought a new insight into molecular mechanisms contributing to the malignant phenotype of PMBCL and this could direct development of targeted therapies.
